# Medical education and research environment in Qatar: a new epoch for translational research in the Middle East

**DOI:** 10.1186/1479-5876-9-16

**Published:** 2011-01-27

**Authors:** Lotfi Chouchane, Ravinder Mamtani, Mohammed H Al-Thani, Al-Anoud M Al-Thani, Marco Ameduri, Javaid I Sheikh

**Affiliations:** 1 Weill Cornell Medical College in Qatar, Education City, P.O. Box 24144, Doha, Qatar; 2 Supreme Council of Health, P.O. Box 42, Doha, Qatar

## Abstract

Recent advances in medical technology and key discoveries in biomedical research have the potential to improve human health in an unprecedented fashion. As a result, many of the Arab Gulf countries, particularly Qatar are devoting increasing resources toward establishing centers of excellence in biomedical research. However, there are challenges that must be overcome. The low profile of private medical institutions and their negligible endowments in the region are examples of such challenges. Business-type government controlled universities are not the solution for overcoming the challenges facing higher education and research programs in the Middle East.

During the last decade, Qatar Foundation for Education, Science and Community Development has attracted six branch campuses of American Institutions of higher learning to the Education City in Qatar, a 2500-acre area, which is rapidly becoming a model of integrating higher education and research in the region. Not-for profit, time-tested education institutions from abroad in public-private partnership with local organizations offer favorable conditions to build robust research programs in the region. Weill Cornell Medical College in Qatar (WCMC-Q) of Cornell University is an example such an institution. It is the first and only medical school in Qatar.

WCMC-Q's interwoven education, research and public health based framework lays a sturdy foundation for developing and implementing translational medicine research programs of importance to the State of Qatar and Middle Eastern nations. This approach is yielding positive results. Discoveries from this program should influence public policy in a positive fashion toward reducing premature mortality and morbidity due to diabetes, obesity, heart disease and cancer, examples of health conditions commonly encountered in Qatar.

## Introduction

A monarchy, Qatar has been ruled by the Al-Thani family since the mid-1800 s. Since its independence in 1971, the nation has undergone remarkable social, economic and industrial development. Recently, the State of Qatar won the bid to host 2022 FIFA World Cup. It is evident that Qatar has transformed itself from a poor British territory into a wealthy oil and natural gas rich state that provides ample growth opportunities for businesses, social events, education and research institutions. According to the Qatar Statistics Authority, on Sept. 30, 2010, there were 1,642,235 Qatari residents, approximately 350,000 of who are Qatari citizens. The remaining residents are expatriates chiefly from South Asia and from non-oil-rich Arab states.

Countries in the Middle East including Gulf Cooperation Council (GCC) nations such as Qatar and United Arab Emirates have experienced a reduction in their mortality rates. In general, life expectancy has increased and people are living longer, many with debilitating non- communicable diseases (NCDs), such as diabetes, cancer and heart disease [1,2].

Health care continues to evolve in the GCC nations. The nations have committed to combating the widespread prevalence of NCDs and the morbidity associated with them [2]. Qatar has been at the forefront of initiating new research, clinical and community projects in controlling these diseases. In general, Qatar's goal is changing from a disease based approach to a more comprehensive evidence based integrative multidisciplinary care and a preventive approach to disease and patient management. Evidence based approach will necessitate developing programs aimed at high quality basic science and public health research with a view to improve the quality of life, and reduce morbidity and premature mortality associated with commonly occurring chronic diseases such as diabetes, obesity and cancer. Education programs, which offer opportunities for research and ideal clinical experience, are required. Developing translational research programs in the Middle East is imperative.

But building a robust, viable research culture in the Middle East is a challenge. There are several reasons for this - one, the Arab world's 200 universities have almost negligible endowments with business and lack adequate venture capital; two, most Arab universities are largely state owned and spend only around one percent of their budgets on research compared to an international average of 35 percent; three, some wealthy countries in the region are lacking in their human capacity building but have funding; and four, low and middle income nations are lacking in financial resources despite having well-educated professionals and scientists [3].

Based on our own collective experience in global health, medical education and research, we feel business-type government controlled universities are not the solution for overcoming the challenges facing higher education and research programs in the Middle East. However, not-for profit, time-tested education institutions from abroad with local financial support and working in close collaboration with the host country's institutions show promise and may offer exciting opportunities. A case in point is Weill Cornell Medical College in Qatar (WCMC-Q). WCMC-Q's interwoven framework of education, research, public health and clinical components lays a sturdy foundation for developing evidence based translational research as discussed in this review.

We begin our review by briefly discussing the educational and research environment. Our discussion continues on Medical Education in Qatar, and provides a summary of student demographics and their interests, and pre-medical and medical education programs at WCMC-Q. This is followed by a brief description of WCMC-Q's public health and research activities. We then summarize the challenges WCMC-Q faces and the opportunities it provides to its faculty and their collaborators. Documenting our experience and the lessons learnt might be instructive to those considering establishing similar programs internationally.

### Research and Education Environment in Qatar

Qatar Foundation (QF), which was established in 1995, is an independent, private, not for profit organization, whose mission is "to prepare the people of Qatar and the region to meet the challenges of an ever-changing world, and to make Qatar a leader in innovative education and research." Under the leadership of His Highness Sheikh Hamad Bin Khalifa Al-Thani, the Emir of Qatar and founder of Qatar Foundation, and Her Highness Sheikha Mozah Bint Nasser Al-Missned, Chairperson of Qatar Foundation, the Foundation is "transforming Qatari society by educating the rising generation to the highest world standards - these will be the skilled professionals who will be the country's future leaders. It is turning Qatar into a producer of knowledge by building a research base. Some of the new ideas will reach the stage of commercialization, helping diversify the economy" [4].

Under the umbrella of QF, there are several premier research and or education institutions. These include the Education City (EC) of which WCMC-Q is an integral part, Qatar Science & Technology Park (QSTP) and the Qatar National Research Fund (QNRF). The overall intent is to connect the industry, academic and government sectors into what is commonly referred to as *the Triple Helix model *[4,5]. The model provides a conceptual framework for regional development.

Education City in Doha, home to six American University branch campuses including Cornell, Georgetown, Texas A&M and Carnegie Mellon, is the flagship of Qatar Foundation. It is spread over 2,500 acres. With the exception of Weill Cornell Medical College of Cornell University, programs offered by the EC universities initially were limited to undergraduate degrees but recently graduate degrees have been initiated by Virginia Commonwealth University and Texas A&M University-Qatar. Additionally, there are plans for EC universities to collaborate with the industry as their research programs mature.

QSTP facilitates the engagement of the private sector with the universities, as a base for multi-national and national companies to establish research centers, and an opportunity for knowledge-based entrepreneurs to create new businesses. It has already attracted tenants such as EADS, Microsoft, ExxonMobil, GE and Shell, the latter of which is to set up a $100 m gas- to-liquids research center. R&D is focused in areas related to the economy of Qatar, such as gas and petrochemicals, healthcare, information and communication technologies, water technologies, the environment and aircraft operations. QSTP also recently announced two venture-capital funds of $130 m to help commercialize local innovations, and the QNRF is providing public funding needed to support basic and applied research.

In accordance with its mission, the Qatar Foundation has embarked on an innovative and visionary set of initiatives to create lasting benefits for the country of Qatar and to increase the visibility of Qatar within the global community. A crucial component of these initiatives is the establishment of infrastructure aimed at improving the health and quality of life of the Qatari population. Weill Cornell Medical College in Qatar's charge includes a leadership role in the effort to address important biomedical research and healthcare needs in Qatar.

**Figure 1:**
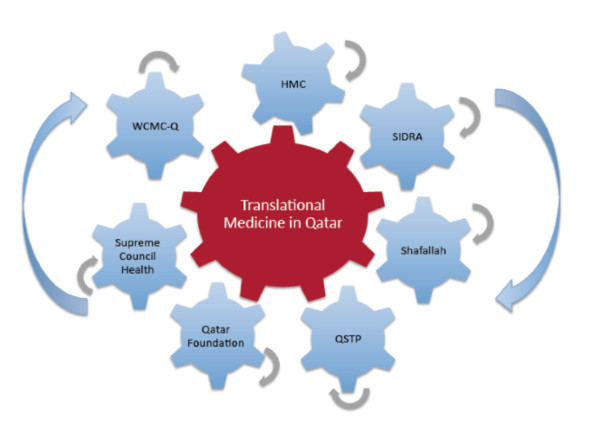
**WCMC-Q's Collaboration with Core Qatar Partner is Key to Advancing Translational Medicine in Qatar**. Each component is a cog in the "central wheel", which represents the Translational Medicine enterprise in Qatar. WCMC-Q: Weill Cornell Medical College in Qatar; HMC: Hamad Medical Corporation; SIDRA: a teaching hospital; Safallah: Special Learning and Research Center for children with disabilities; QSTP: Qatar Science and Technology Park.

The main focus of Qatar Foundation' s mission is a partnership building approach which allows institutions in Qatar with similar objectives in medical education, research, public health and healthcare to come together: WCMC-Q and its US-based sister institution WCMC-NY, Hamad Medical Corporation (HMC), Sidra Medical and Research Center, QSTP, Supreme Council of Health (SCH), and QF the Qatar Foundation (Figure 1). Qatar's commitment to research is evident in many reports and comments of scientists from around the world [6].

### Medical Education

WCMC-Q, a branch campus of Weill Cornell Medical College in New York (NY), is the unique medical school in Qatar. It is located in Doha, Qatar, about 11,000 KM distant from its parent campus in New York. WCMC-Q is housed in Education City. WCMC-Q awards the same MD degree as the main campus in New York.

This institution, its students, faculty, educational, clinical and research resources, processes and traditions are in the early stages of development. WCMC-Q graduated its inaugural class in May 2008. Its students and faculty, as well its local affiliate Hamad Medical Corporation (HMC) faculty, are remarkably diverse in terms of their cultural, social and educational backgrounds.

WCMC-Q currently offers three separate educational programs: a) two year premedical program, b) four year medical (MD) program and c) one-year foundation program (primarily aimed at Qatari students), which provides intensive training to high school graduates in science, math and English to better prepare them for the pre-medical program.

#### WCMC-Q medical student demographics and interests

Tables 1 and 2 show the demographics of the current student body (Foundation, Pre-medical and Medical Programs), which are composed of students from 36 different countries with Qatari nationals constituting 18% of the student body. The male and female percentage distribution of students is about 54 and 46 respectively.

**Table 1:** Distribution of Medical, Premedical and Foundation Students by Gender (Numbers as of Sep. 2010)

	Total Number	Females	Males
**Medical Students**			

**Class 2011**	38	20	18

**Class 2012**	28	7	21

**Class 2013**	40	22	18

**Class 2014**	42	18	24

**Total Medical Students**	**148**	**67**	**81**

**Pre-medical Students**	**109**	**52**	**57**

**Foundation**	**17**	**8**	**9**

**All Students**	**274**	**127**	**147**

**Percent distribution**		**46%**	**54%**

**Table 2:** Distribution of Students by Citizenship. (Numbers as of September 2010)

Nationalities	Count	Percent
Qatar	48	18

Egypt	32	12

United States	29	10

India	24	9

Jordan	18	6

Syria	16	6

Canada	15	5

Lebanon	14	5

Iraq	13	5

Pakistan	13	5

Bahrain	6	2

Sudan	5	2

France	4	1

Oman	4	1

Others	33	12

**Total Number of Students**	**274**	**100**

Other countries include: Australia, Bangladesh, Yemen (3 each); Republic of Korea (South), Palestine-Egypt, Palestine-Lebanon, Russia, Saudi Arabia, Sri Lanka (2 each); Algeria, Bosnia, Germany, Kenya, Kuwait, Mauritania, Mauritius, Nepal, Philippines, Tanzania, Tunisia, U.A.E., United Kingdom (1 each).

Table 3 displays the numbers of graduating students in years 2008, 2009 and 2010, and those who conducted research under supervision of at least 8 weeks in duration during their four years of medical education training at WCMC-Q. The table also shows the numbers of students who engaged in local/global public and other volunteer activities. It is noteworthy that 53 to 100% of graduating students engaged in research under supervision. As is evident from the table there is also an increasing student interest in global health activities. Our students have traveled to India, Nepal, Tanzania and Haiti to gain this type of experience.

**Table 3:** Numbers of graduating students, who gained research and other experiences

		Number(%) of students gaining certain experience		
**Graduating Class**	**Number**	**Research**	**Local public Health/Community**	**Global Health**

Class of 2008	15	10 (66)	8 (53)	3 (20)

Class of 2009	17	9 (53)	9 (53)	4 (23)

Class of 2010	17	17 (100)	10 (59)	8 (47)

Research experience - is at least 8 weeks of approved and supervised research experience, which the students gained during their medical school education program.

Local public health - is at least the equivalent of 2 weeks of any volunteer local health related experience or any other volunteer work which contributed to improving the quality of life of people living in Qatar.

Global health experience - is at least the equivalent of 2 weeks of any supervised voluntary or for credit experience (as an elective) in countries other than the US and Qatar.

#### WCMC-Q education programs

##### Pre medical education

The Pre medical education program at WCMC-Q is a flexible two or three-year program to which students are admitted following their high school education. Most students take the two-year option with condensed mathematics and sciences courses. For those students coming from a disadvantaged high school background or in need of development in their English skills, a one-year Foundation Program has been added before the premedical program. The Foundation Program offers pre-college courses in the sciences and an English as a Second Language (ESL) course, along with a focus on developing study skills and professionalism. This is quite different from the typical situation in the US, where, barring very few exceptions, all students entering a medical school have completed a four-year undergraduate degree.

The Premedical Program at WCMC-Q offers a range of courses chosen to meet WCMC Q admission requirements and to offer breadth of education. While most of the courses focus on mathematics, physics, chemistry, and biology, an effort has been made to offer humanities and social science courses such as psychology and medical ethics. While such a curriculum may appear rigid and too heavily science-oriented, it allows for a solid and integrated learning experience. There is a close and continuous interaction among the faculty delivering the courses, and the students have the opportunity to better appreciate the unifying themes and concepts lying behind the nominally distinct sciences.

Additionally, premedical students have the opportunity to participate in research projects under the guidance of premedical, medical, and research faculty. The Premedical Program has been very successful in producing student capable to enter the Medical Program and to perform at the high level there required.

##### Medical program

WCMC-Q and NY use the same curriculum and learning objectives. The curriculum, which integrates basic with clinical sciences, is progressive, challenging and rigorous. It engages students in active learning, self-directed inquiry, and small group discussions. These methods are integrated with seminars and lectures provided by faculty from WCMC-Q, NY, and Hamad Medical Corporation (HMC), an affiliate of WCMC-Q.

The medical curriculum is designed to provide students a series of integrated, interactive courses. The first and second year basic science curriculum consists of five courses and an introduction to clinical skills. These courses are - Molecules Genes & Cells, Human Structure & Function, Host Defenses, Brain and Mind, and Basis of Disease. There are two additional clinical based courses, Medicine Patients & Society I and II, which the students must complete before beginning their clinical experience in the third year. The third and fourth year clinical curriculum requires completing several required core clinical clerkships and electives, and one course, Advanced Basic Science. The students complete their core clerkships in medicine, primary care, neurology, obstetrics and gynecology, pediatrics, psychiatry, and general surgery at HMC affiliates in Qatar. Additionally, almost all the students spend approximately 12 weeks at New York Cornell Presbyterian - Cornell and affiliated hospitals where they complete sub-internships and electives. The clerkship sites in Qatar, developed in collaboration with HMC, include Hamad General Hospital (in-patient, ER and outpatient clinics), Women's Hospital, Shafallah Center, Primary Health Centers (PHC) and other local hospitals and centers. Students also complete a two-week required clerkship in public health and another short course, Medicine, Patients and Society III aimed at promoting humanistic practice. The public health course encourages working in teams and building partnerships, which promote coordination of written and oral communication skills. These skills are vital to public health professionals and researchers.

WCMC- Q's program prepares its students exceptionally well. This is reflected in their performance on the standardized test, namely the United States Medical Licensing Examination (USMLE), which is a three-part examination for medical licensure in the United States and is sponsored by the Federation of State Medical Boards (FSMB) and the National Board of Medical Examiners (NBME). Here we report on the performance of the students who took the USMLE Step 1 and II examinations for the first time in the period 2006 - 10. As can be seen from the Table 4 the USMLE I passing rate of WCMC-Q students is 86% as compared to 93% for the US students. This difference is not statistically significant. Table 5 shows the USMLE II passing rate for both the US and WCMC-Q students is 96%.

**Table 4:** Performance of Examinees Taking USMLE Step I for First Time in The Years 2006, '07 and '08 (Students from the Classes of 2008, 09 and 10)

	WCMC-Q*	US**
Number Tested	54	55604

Number Passing	47	51947

Percent Passing	87	93

P value = 0.059 (Pearson chi square); 0.08 (Fisher 's exact)

**Table 5:** Performance of Examinees Taking USMLE Step II Clinical Knowledge (CK) for First Time in The Years 2007-08, '08-09 and '09-10 (Students from the Classes of 2008, 09 and 10)

	WCMC-Q*	US**
Number Tested	45***	53505

Number Passing	43	51525

Percent Passing	96	96

P value = 0.683 (Fisher's exact)

*Several students from the Classes of 2008, 09 and 10 have/had taken leave of absence for personal reasons or for pursuing research. Therefore the number of students who were tested that appear in the Tables 4a and 4b do not match with the numbers of graduating students in the Table 3

**Source: Weill Cornell Medical College, Registrar's Office.

***There were three additional students who passed their USMLE CK; however, our Office does not have a record of the dates when they took the examination, and therefore, are excluded from the number tested.

WCMC-Q gradating class of 2010 demonstrated the high quality of their education by being able to successfully compete and secure residency spots in their fields of interest at excellent institutions in the US (See Table 6). Out of 17 graduating students, 11 (65%) are pursuing postgraduate residency training in the US. Four (23%) have decided to take research fellowships at US institutions and the remaining two (12%) have opted for postgraduate education at HMC. These results reflect very positively on the quality of the WCMC-Q program leading to the M.D. degree.

**Table 6:** Residency Programs at which Class 2010 Graduates are pursuing their clinical training*

	Program	Specialty	Location
1	Vanderbilt Univ Med Ctr- TN	Internal Medicine	Nashville, Tennessee - USA

2	Providence Hospital & Medical Centers	Internal Medicine	Southfield, Michigan - USA

3	George Washington University Medical Ctr	Internal Medicine	Washington DC - USA

4	Hamed Medical Corporation	General Surgery	Doha - Qatar

5	Methodist Hospital System	Internal Medicine	Houston, Texas - USA

6	NYP Hospital - Weill Cornell Med Ctr	Internal Medicine	New York, New York - USA

7	Johns Hopkins Hospital	Internal Medicine	Baltimore, Maryland - USA

8	University of Louisville School of Medicine	Obstetrics-Gynecology	Louisville, Kentucky - USA

9	North Shore-Long Island Jewish Hlth System	General Surgery	Long Island, New York - USA

10	Drexel University COM/Hahnemann Univ Hosp	Obstetrics-Gynecology	Philadelphia, Pennsylvania - USA

11	Hamed Medical Corporation	Anesthesiology	Doha - Qatar

12	Cleveland Clinic Foundation	Internal Medicine	Cleveland, Ohio - USA

13	University at Buffalo School of Medicine	Pediatrics	Buffalo, New York - USA

* There are four additional students who are pursuing their research interests at institutions in the US.

Research is growing at WCMC-Q and will provide many opportunities to our students in years ahead. While opportunities for off site student field placements are limited, our partnerships with government and health care institutions are beginning to produce positive results.

### Public Health Agenda and Activities

WCMC-Q is committed to working with local public health and global partner institutions that will be most appropriate at this stage to advance most effectively the mission of WCMC-Q in education, research, and patient care, as well as population well being of people in the State of Qatar.

In this context WCMC- Q is embarking on the following public health agenda in the three areas of education, research and community related matters:

A. Education: to strengthen and augment existing educational activities; and develop and implement new programs. Examples of these include a) student exchange programs and b) courses and programs related to disciplines such as research methodology, public health, nanotechnology, nutrition, bio-informatics, and public health.

B. Research: to expand and increase collaborative global and local research initiatives especially on topics of public health importance such as obesity and motor vehicle accidents. We will increase public health research on projects of relevance to the local communities in Qatar. This will be done in close cooperation with the Department of Public Health, Supreme Council of Health (SCH), Qatar and other stakeholders.

C. Community and related matters: to enhance community, health awareness and patient care related services that support the needs of people in Qatar.

### Biomedical and Translational Research

**Figure 2:**
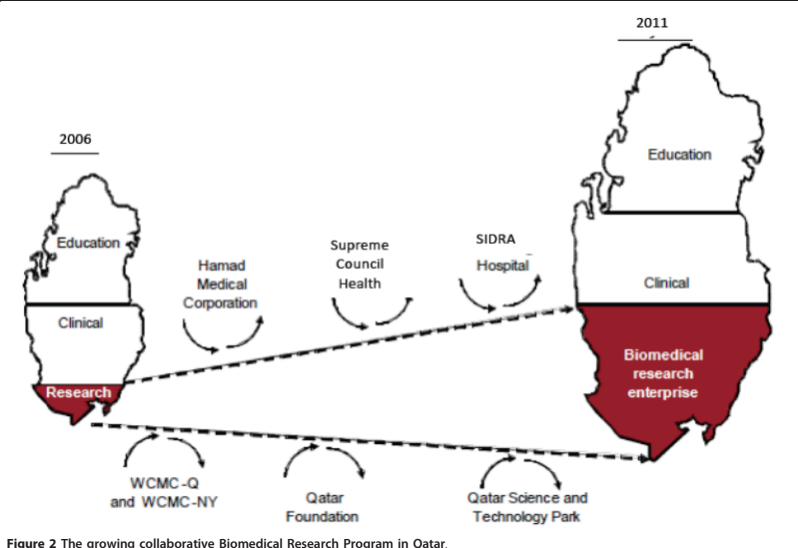
**The growing collaborative Biomedical Research Program in Qatar**.

WCMC-Q's research program aims to a) build a self-sustaining core of top biomedical scientists by recruiting, retaining, and training top talents, and b) establish strong research programs in Qatar which target important public health problems and healthcare issues. WCMC-Q research program is consistent with the State of Qatar's strategy on education, research, community development and health care (Figure 2).

### Challenges

WCMC-Q has made excellent progress in establishing a world-class research enterprise located in Qatar conducting cutting-edge biomedical research. This enterprise is attracting, retaining and developing research talent. The College's project is work in progress. It will contribute to the ground-breaking scientific ideas and allow for appropriate commercialization of research findings with QSTP. The creation of such an enterprise is a long-term endeavor that faces many challenges, examples of which include: 1) the challenge faced by translational medicine, which is the difficulty in truly being a trans-disciplinary science that brings together researchers and practitioners that traditionally work within their own "silos" of practice [7], 2) the creation of sustainable research infrastructure, 3) building a strong research community, 4) recruiting and retaining top-notch faculty and researchers, and 5) lack of recognition of Qatar as a core member of the global research community. Despite these challenges Qatar has made considerable progress and initiated many projects, which will lay solid foundation for effective clinical and preventing strategies in combating NCDs. These strategies will not only reduce the incidence of these diseases but also reduce pain and alleviate suffering associated with them. An outline of QF's and WCMC's projects and other initiatives appears below.

### Opportunities

There are many research opportunities available to the students and faculty at WCMC- Q. An example of such opportunities is the availability of research funds through the Qatar Foundation's Qatar National Priority Research Program (NPRP). This program funds meritorious proposals ranging from US$ 20,000 up to US$ 350,000 per year for a duration of one, two or three years. The program encourages local and international collaboration. More recently, QNRF launched a new program, called National Priorities Research Program - Exceptional Proposals (NPRP-EP). This new program seeks investigators with proposals of high merit, which require extra funds and more time for their completion. The program provides up to US $ 5 million per project for a maximum period of five years.

In continuing to nurture various opportunities, and develop and implement the translational research program, WCMC-Q will be guided by the following objectives:

1. Using bench-to -bedside research approach in addressing Qatar's major health problems such as diabetes, cancer, obesity and heart disease.

2. Building local research capacity by establishing sustainable training programs/courses for students and physicians.

3. Developing and nurturing viable, collaborative partnerships with local and international institutions to further enhance research-building capacity.

4. Establishing an Institute for Global and Public Health, which will engage in research that can positively influence public policy so as to address major health problems such as obesity and motor vehicular accidents.

Reflected in WCMC-Q objectives are several integrated translational medicine research proposals that have been developed and funded by Qatar Foundation's Qatar National Priority Research Program (NPRP). Others have been submitted for funding to the same agency. We present synopsis of three of such proposals.

#### 1. Genomics and proteomics of breast cancer in Arab populations

The main goal of this project is to address key questions of the nature of genetic predisposition and protein biomarkers for certain types of breast cancer particularly frequent in Arab populations and to translate that to clinical management, including diagnosis, prevention and therapeutics. It aims to establish excellence in the Middle East/North Africa region in the cancer research field, which could be an instrument to tackle the fragmentation of cancer research in the Arab countries.

#### 2. Public health and genomic aspects of obesity in Qatar

This multidisciplinary project aims to identify and understand the a) epidemiologic risk factors of obesity, b) the functions and interactions of macromolecules in cells and c) decipher the biological mechanisms of obesity among Qataris. The study findings will be used in developing novel strategies in the treatment and prevention of obesity in Qatar and other nations in the region.

#### 3. Nanotechnologies and treatment of obesity

This project explores the significance of nanotechnological approach in the treatment of obesity. The results of this project will play a fundamental role in setting the stage for major programs in Nano-Medicine and Stem Cell-Based therapies and technologies in Qatar, as well as the translation of the scientific discoveries from such programs in predictive medicine for the prevention and treatment of obesity and metabolic diseases.

We should comment on one other translational research development. Given the high prevalence of diabetes and obesity in Qatar, WCMC-Q is establishing new Diabetes, Obesity and Metabolic Syndrome centre (DOMS). The DOMS Center's ultimate vision is to create a solid infrastructure, which supports the growth of collaborative and multidisciplinary research initiatives in Qatar. The Center's state of the art facilities dealing with genomics, proteomics, imaging, and computational and health quantitative sciences will be available to the scientists for their research projects. The Center will also develop educational and training programs, and partner with Supreme Council of Health on topics of public health importance to the country and the region.

The above multidisciplinary projects with national and global partners have investigators from different backgrounds. The research findings from these projects have the potential of significantly improving the treatment, management, and prevention of commonly occurring non-communicable diseases such as diabetes, cancer and obesity. The findings will also help in the development and implementation of population based health promotion programs.

## Conclusion

Promising collaborative multidisciplinary translational research as illustrated in this review is an encouraging development in Qatar and its neighboring GCC nations by extension. WCMC-Q's interwoven education, research and public health based framework provides a robust platform for translational medicine research programs. This approach is yielding positive results. Discoveries from this program should influence public policy in a positive way. Our approach encourages local and global collaboration and partnership with investigators and research institutions from around the world. Our research initiatives have sparked optimism among public health officials, clinicians, and researchers to fully seize the new opportunities in reducing premature mortality and morbidity associated with NCDs such as diabetes, cancer, heart disease and obesity. We feel many studies that are under way in Qatar will provide promising prevention strategies and life saving treatments for the people in the State of Qatar and its neighboring nations.

## Competing interests

The authors declare that they have no competing interests.

## Authors' contributions

LC and RM conceived the manuscript and its design; provided detailed ideas and discussions, and contributed to the manuscript writing on the introduction, medical education program, and challenges and opportunities sections of the article. LC contributed to manuscript writing of the research environment in Qatar. RM contributed to the manuscript writing of the public health and student data components of the article. MA conceptualized and wrote the premedical education component of the manuscript. MHA and AMA - provided ideas and discussions on the history of Qatar, Qatar Foundation and the opportunities section of the article. JIS made intellectual contributions; participated actively in revising the final draft of the manuscript and contributed in figures. All authors read and approved the final manuscript.
